# Genotypic virulence profiles and mobile genetic elements related to invasiveness in *Neisseria gonorrhoeae* strains

**DOI:** 10.1590/S1678-9946202668025

**Published:** 2026-03-13

**Authors:** José Victor Bortolotto Bampi, Saidy Vásconez Noguera, Sania Alves dos Santos, Ana Paula Marchi, Marina Farrel Côrtes, Joyce Vanessa da Silva Fonseca, Ivan Lira dos Santos, Flávia Rossi, Maria Luiza Bazzo, Gwenda Hughes, Igor Borges, Silvia Figueiredo Costa

**Affiliations:** 1 Universidade de São Paulo, Faculdade de Medicina, Departamento de Moléstias Infecciosas e Parasitárias, São Paulo, São Paulo, Brazil; 2 Universidade de São Paulo, Faculdade de Medicina, Instituto de Medicina Tropical de São Paulo, São Paulo, São Paulo, Brazil; 3 Pontifícia Universidade Católica de Campinas, Faculdade de Medicina, Campinas, São Paulo, Brazil; 4 Universidade de São Paulo, Faculdade de Medicina, Hospital das Clínicas, Divisão de Laboratório Central, Serviço de Microbiologia Clínica, São Paulo, São Paulo, Brazil; 5 Universidade Federal de Santa Catarina, Florianópolis, Santa Catarina, Brazil.; 6 London School of Hygiene and Tropical Medicine, Department of Infectious Disease Epidemiology, London, United Kingdom; 7 Centres for Antimicrobial Optimisation Network, São Paulo, São Paulo, Brazil

**Keywords:** Neisseria gonorrhoeae, Invasive, Non-invasive, Virulence, Whole genome sequencing

## Abstract

*Neisseria gonorrhoeae* (NG) has become an increasing global health threat due to drug resistance and potentially invasive disease. In this study, we assessed the genotypic profile in both invasive and non-invasive NG strains from patients at University of Sao Paulo Faculty of Medicine Clinics Hospital, Brazil. We analyzed the NG genotypic profiles and clinical data from 25 patients hospitalized at Hospital das Clinicas da Faculdade de Medicina da Universidade de Sao Paulo (HC-FMUSP) from January 2017 to March 2020. A total of 25 isolates underwent whole genome sequencing, of which 10 (40%) were collected from sterile sites like blood and classified as invasive. Invasive and noninvasive specimens were clustered in 5 STs and 11 STs, respectively, sharing 3 STs in common. We identified resistance markers for macrolides (*mtrR*) in 40% of isolates and 92% exhibited at least one resistance marker for beta-lactams (*penA, bla*_TEM-1B_, or *bla*_TEM-135_), predominantly PBP1 and PBP2 alterations. Resistance genes *bla*_TEM-1B_ (7%vs.50%, *p*=0.023) and *tetM* (13%vs.80%, *p*=0.002) were more frequently found in invasive disease isolates, always accompanied by the mobile genetic elements pJD4 and pEP5289, respectively. Associated with these elements were two toxin encoding genes (*vapD* and *zeta*1/2) previously described as possible virulence factors and resistance genes for beta-lactams and tetracyclines. Results revealed that invasive strains of NG are genotypically clustered and frequently harbor two plasmids which may be associated with invasive disease. Resistance markers for beta-lactam as PBP1 and PBP2 alterations warn against a future potential resistance to this class.

## INTRODUCTION

Sexually transmitted infections (STIs) are a global public health issue and can be associated with severe outcomes^1-4^. Estimates indicate that approximately 1 million STIs are contracted worldwide each day, with about 82.4 million *Neisseria gonorrhoeae* (NG) infections occurring globally in 2020. According to the World Health Organization (WHO), the Americas registered approximately 9.8 million NG cases during this period^3^. In response to this global public health concern, the WHO has set targets to reduce the incidence of NG infections by 2030 as part of its Global Health Strategy for STIs.

Treating bacterial infections, especially those caused by NG, is challenging due to increasing antimicrobial resistance^5^. *Neisseria gonorrhoeae* has shown resistance to all classes of antimicrobials available for treatment, which may render it untreatable^6-8^. In this scenario, knowledge about the resistance and virulence profile of infectious agents can play a crucial role in formulating effective public health policies to combat STIs^1^.

Without early diagnosis and adequate treatment, NG infections can lead to complications such as pelvic inflammatory disease, ectopic pregnancy and infertility^9-11^. Less frequently, untreated disease could manifest as invasive gonococcal disease (IGD) where the bacteria are isolated from sterile sites due to hematogenous spread from the initial mucosal infection focus. IGD accounts for 0.5% to 3% of gonorrhea cases and can manifest as purulent arthritis, endocarditis, meningitis, osteomyelitis, and bloodstream infection^11^.

In addition to host-related factors like immunosuppression^12^, research has already linked a higher likelihood of IGD presentation to genotypic elements of NG strains. Studies have shown gonococcal genetic islands associated with conjugation mechanisms, *opa* genes related to phagocytosis resistance, allele 1A of the *porB* gene encoding porin variations, among others^13,14^.

In Brazil, studies on the genetic characterization of NG isolates and virulence profiles are limited and primarily focused on antibiotic resistance surveillance^15-19^. Despite their scarcity, the available data indicate a high and increasing antimicrobial resistance in NG, with resistance rates of 67.3%, 40.0% and 10.6% for ciprofloxacin, tetracyclines and azithromycin, respectively. To date, no Brazilian strains with ceftriaxone resistance have been described in surveillance studies^18^.

Thus, this study assessed the genotypic virulence profiles related to IGD compared with non-invasive NG strains from individuals with gonococcal disease hospitalized at the Hospital das Clinicas da Faculdade de Medicina da Universidade de Sao Paulo (HC-FMUSP).

## MATERIALS AND METHODS

### Study population

This retrospective study included 25 NG samples from all available isolates of invasive and non-invasive gonococcal disease cases treated at the HC-FMUSP from 2017 to 2020.

### Ethics

Ethics approval for this study was obtained from the HC-FMUSP Ethical Committee (Sao Paulo, Brazil), Nº 5.054.730.

### Patient data

Patients were evaluated by reviewing epidemiological and clinical data registered on electronic medical records. Clinical and demographic variables, including age, gender, date and isolation site, use of immunosuppressive medications, serology for other STIs, treatment details, and hospitalization status were extracted. Additional patient data were recovered from the Ministry of Health's Laboratory Exams Control System (SISCEL) to access records in the national HIV treatment program.

### Bacterial isolates and genomic analysis

A total of 25 NG isolates from invasive (n=10) and non-invasive (n=15) infections were identified by matrix-assisted laser desorption/ionization time-of-flight (MALDI-TOF; Bruker Biotyper 3.1, Bruker Daltonics) available at the HC-FMUSP. All isolates underwent Whole Genome Sequencing (WGS) by Ion Torrent PGM technology (Thermo Fisher Scientific, Waltham, MA, USA) located at the Sao Paulo Institute of Tropical Medicine^20^.

Resistome was analyzed using ResFinder 4.0 and Comprehensive Antibiotic Resistance Database Card (CARD) 4.0^21,22^. Virulence genes were identified using the Virulence Factor Database (VFDB) tool^23^. NG Multi-Antigen Sequence Typing (NG-MAST) and Sequence Typing for Antimicrobial Resistance (NG-STAR) were performed and types identified by the PubMLST tool^24^, also confirming species identification. Mutations in resistance genes were manually searched in the genomes using the Basic Local Alignment Search Tool (BLAST). Creating the consensus sequence for *Neisseria gonorrhoeae* involved the assembly of sequences on Unipro UGENE 48.1, using a closely related reference sequence (sequence typing) as a key component^25,26^.

A phylogenetic tree was constructed using the REALPHY tool (v. 1.12). *Neisseria gonorrhoeae* FA19 (GenBank accession Nº NZ_CP012026.1) served as reference for molecular characterization. Single Nucleotide Polymorphisms (SNPs) were calculated on Seaview^25^, and multiple sequence alignments were recreated using PhyML with 500 bootstrap replications for tree construction^27^. All *Neisseria gonorrhoeae* sequencing data from this study were submitted to GenBank and are available through Bioproject Nº PRJNA625087.

### Data analysis

Categorical variables were expressed as absolute numbers (percentage), and continuous variables as medians (interquartile range [IQR]). Comparison of categorical variables used the chi-squared test or Fisher's exact test as appropriate. Statistical tests were two-tailed, with a significant level of 0.05. All analyses were performed on Stata software (version 13.0, StataCorp LLC, Texas, USA).

## RESULTS

Analysis included a total of 25 patients (Table 1) who sought hospital care due to NG related symptoms. Of these, 60% were male and 64% were white, with a median age of 23 years (p25=20, p75=32). Three (12%) patients were undergoing treatment with an immunosuppressive medication (Mycophenolate + Prednisone, Hydroxychloroquine + Methotrexate + Prednisone, and Mycophenolate) for Systemic Lupus Erythematosus (SLE).

**Table 1:** Characterization of the 25 individuals with *Neisseria gonorrhoeae* at the Clinics Hospital, Brazil

Variables		N = 25
**Age**		23 (20; 32)
**Male sex**		15 (60%)
**Ethnicity**		
	White	16 (64%)
	Indigenous	1 (4.0%)
	Black	2 (8.0%)
	Unspecified	4 (16%)
	Asian	2 (8.0%)
**Use of immunosuppressive drugs**		3 (12%)
**Serology test for STIs**		14 (56%)
	HIV	14 (56%)
	Hepatitis B	13 (52%)
	Hepatitis C	13 (52%)
	Syphilis	12 (48%)
**Hospitalization**		12 (48%)
**Invasive gonococcal disease**		10 (40%)
**Isolation site**		
	Anogenital	7 (28%)
	Articular	7 (28%)
	Blood Culture	3 (12%)
	Ophthalmic	8 (32%)

STIs = Sexually transmitted infections.

Serological tests for other STIs (HIV, Hepatitis B, Hepatitis C, and Syphilis) were requested for only 14 (56%) patients; testing for *Mycoplasma, Chlamydia* or other infections was not performed. No patients had positive serology for other STIs, and none were registered in the national HIV treatment program. Twelve (48%) patients required hospitalization for investigating and treating complications, with no recorded fatalities.

Isolates were obtained from anogenital (n=7; 28%), ophthalmic (n=8; 32%), joint (n=7; 28%), and blood culture samples (n=3; 12%). Ten (40%) IGD cases were identified (Table 1). One patient in the blood culture group appeared in medical records as having aortitis due to NG, while another was diagnosed with endocarditis on a prosthetic pulmonary valve.

Four patients (16%) left hospital care without receiving antibiotic therapy, all of whom had presented with anogenital symptoms. Of the 21 remaining patients, 18 (85%) received ceftriaxone as part of their treatment regimen. One patient with ophthalmic symptoms was administered topical ciprofloxacin treatment, while another with anogenital symptoms had been prescribed oral ciprofloxacin. One patient who had presented with joint symptoms was initially treated with clindamycin and gentamicin; seven days after culture results, their treatment was switched to oral azithromycin.

**Figure 1:**
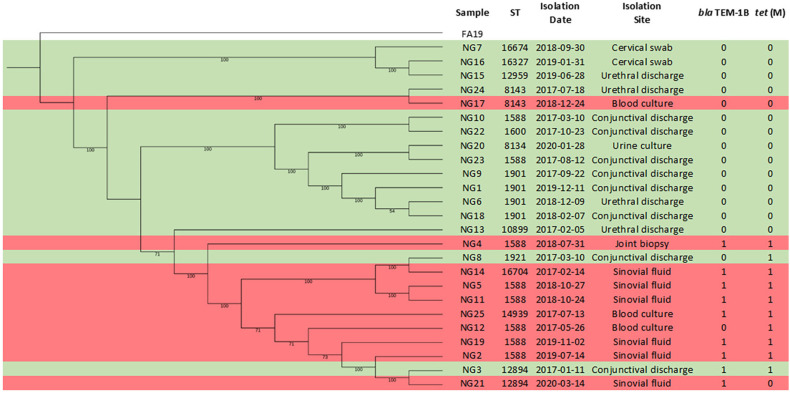
Phylogenetic tree of the analyzed samples with corresponding MLST, isolation site, and presence (1) or absence (0) of the *bla*_TEM-1B_ and *tetM* genes. MLST: Multilocus Sequence Typing; NG: *Neisseria gonorrhoeae*. Red: Invasive Gonococcal Disease (IGD); Green: Non- Invasive Gonococcal Disease (Non-IGD).

Molecular characterization of the isolates identified 13 different Sequence Types (STs), namely: 1588, 1600, 1901, 1921, 8134, 8143, 10899, 12894, 12959, 14939, 16327, 16674, 16704, and four (10899, 16327, 16674, 16704) which had not previously been described in Brazil. STs 1588 (n=8; 32%) and 1901 (n=4; 16%) were the most frequently observed. Phylogenetic tree analysis showed two clusters broadly grouping by ST, differentiating between IGD and non-IGD isolates (Figure 1). Table 2 presents a more detailed description of genotypic profile and individual patient characteristics.

**Table 2:** Descriptive characteristics and genotypic profile of the 25 individuals with *Neisseria gonorrhoeae*

Sample	Invasive	MLST	NG-STAR	Gender	Immunosuppression	Hospitalization	Disease manifestation
NG1	No	1901	1025	Male	No	Yes	Conjunctivitis
NG2	Yes	1588	2088	Female	No	Yes	Septic arthritis, vaginal discharge
NG3	No	12894	2088	Male	No	No	Conjunctivitis
NG4	Yes	1588	957	Male	No	Yes	Septic arthritis, right knee
NG5	Yes	1588	1096	Male	Yes	Yes	Polyarthritis, lumbar abscess
NG6	No	1901	2086	Male	No	No	Urethritis
NG7	No	16674	2086	Female	No	Yes	Vaginal discharge
NG8	No	1921	2019	Male	No	No	Conjunctivitis
NG9	No	1901	90	Female	No	No	Conjunctivitis, keratitis
NG10	No	1588	3155	Female	No	Yes	Conjunctivitis
NG11	Yes	1588	1096	Male	No	No	Polyarthritis
NG12	Yes	1588	2067	Male	No	Yes	Polyarthritis, aortitis
NG13	No	10899	3627	Male	No	No	Urethritis
NG14	Yes	16704	2019	Male	No	Yes	Septic arthritis
NG15	No	12959	2086	Male	No	No	Urethritis
NG16	No	16327	1128	Female	No	No	Vaginal discharge
NG17	Yes	8143	426	Female	Yes	Yes	Polyarthritis
NG18	No	1901	1025	Male	No	No	Conjunctivitis
NG19	Yes	1588	1354	Female	Yes	Yes	Septic arthritis, left ankle
NG20	No	8134	63	Male	No	No	Urethritis
NG21	Yes	12894	1238	Male	No	Yes	Septic arthritis, left knee
NG22	No	1600	121	Female	No	No	Conjunctivitis, keratitis
NG23	No	1588	57	Female	No	No	Conjunctivitis
NG24	No	8143	426	Male	No	No	Urethritis
NG25	Yes	14939	1003	Female	No	Yes	Endocarditis

MLST = Multilocus sequence typing; NG -STAR = *Neisseria gonorrhoeae* sequence typing for antimicrobial resistance; MLST and NG-STAR profiles determined by the PubMLST online tool.

Resistome analysis detected the *rpsJ* and *tetM* genes in 40% of isolates, both associated with tetracycline resistance, whereas 92% of strains exhibited at least one resistance marker gene for beta-lactams (*penA, bla*_TEM-1B_, or *bla*_TEM-135_), predominantly PBP1 and PBP2 alterations. We also identified resistance markers for fluoroquinolones (*gyrA* and *parC*) in 32% and for macrolides (*mtrR*) in 40% of patients (Table 3).

**Table 3:** Descriptive/demographic variables and genes evaluated in bivariate analysis comparing IGD and Non-IGD in 25 individuals with *Neisseria gonorrhoeae*.

Variables		Non-IGD, N = 15	IGD, N = 10	*p*-value^a^
**Age**		23 (22, 31)	22 (20, 32)	0.600
**Gender**				1
	Male	9 (60%)	6 (60%)	
	Female	6 (40%)	4 (40%)	
**Use of immunosuppressive drugs**		0 (0%)	3 (30%)	0.052
**Resistance genes**				
	*bla*_TEM1B_	1 (7%)	5 (50%)	0.023
	*bla*_TEM-135_	1 (7%)	1 (10%)	1
	*penA*	1 (7%)	1 (10%)	1
	Porin PIB	1 (7%)	0 (0%)	1
	*gyrA*	2 (13%)	1 (10%)	1
	*tetM*	2 (13%)	8 (80%)	0.002
	*mtrR*	4 (27%)	4 (40%)	0.667
	*parC*	6 (40%)	1 (10%)	0.179
	PBP1	5 (33%)	4 (40%)	1
	PBP2	13 (87%)	10 (100%)	0.500
**Invasive genes**				
	Opa	6 (40%)	3 (30%)	0.691
	OpC	10 (67%)	9 (90%)	0.345
**Iron absorption genes**				
	*fbpABC*	15 (100%)	9 (90%)	0.400

IGD = Invasive gonococcal disease;

a Fisher's exact test / Chi-squared test.

Other identified genes related to adhesion (LOS and Type IV Pili), efflux pump (FarAB and MtrCDE), IgA protease, immunomodulation (NspA and fHbp), invasion, iron uptake (HmbR, HpuAB, Lbp, Tbp), and stress proteins (KatA, MntABC, MsrAB, RecN). None of the strains exhibited genes related to anti-phagocytosis.

Bivariate analysis showed a marginal association between the use of immunosuppressive drugs and presence of IGD (0% vs. 30%, *p* = 0.052). No demographic and clinical data or virulence gene identified in the database tools exhibited a statistically significant association with IGD. Regarding resistance genes, the *bla*_TEM-1B_ (7% vs. 50%, *p* = 0.023) and *tetM* genes (13% vs. 80%, *p* = 0.002) were more frequently detected in isolates from IGD patients (Table 3). These genes encode for beta-lactamases and modify the binding site of tetracyclines, respectively. All samples exhibiting these genes belonged to the cluster with predominantly IGD cases in the phylogenetic tree (Figure 1).

**Figure 2:**
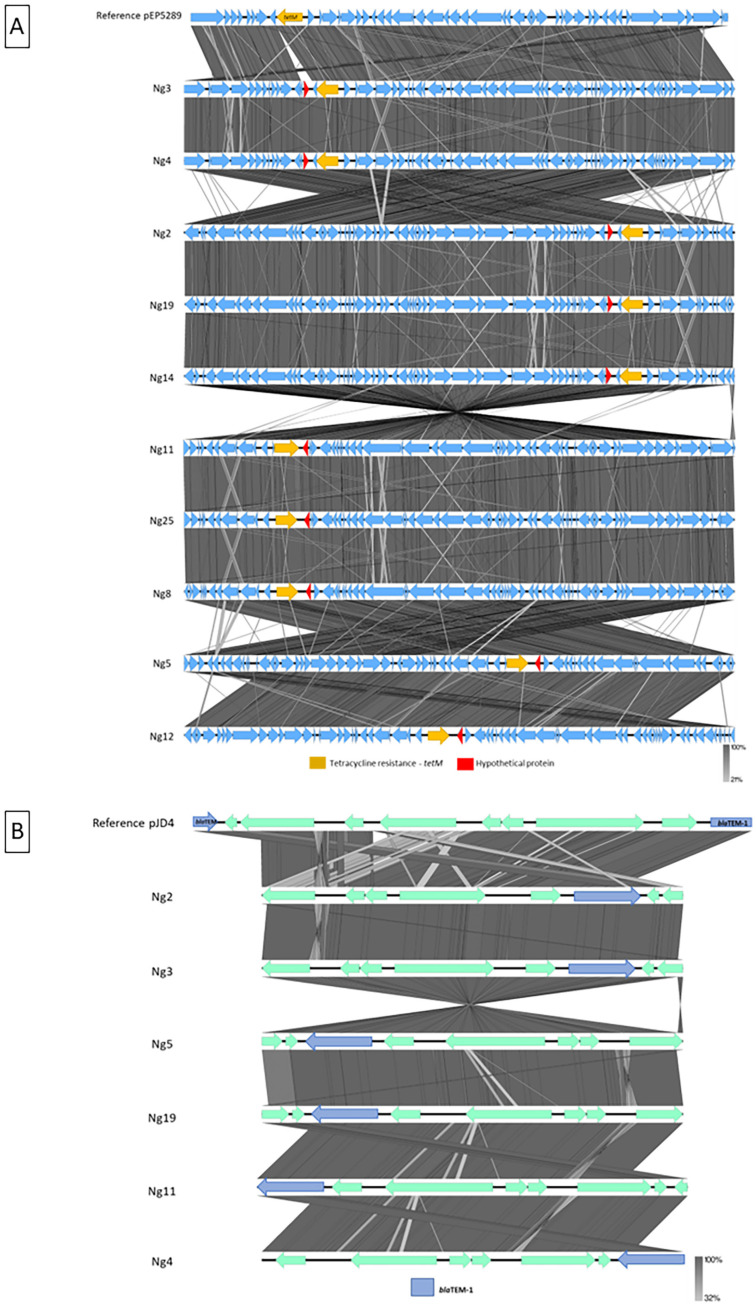
(A) Alignment with reference plasmid pEP5289 with those found in the samples. Yellow: *tetM* gene; Red: Hypothetical protein; Blue: Remaining genes. (B) Alignment with reference phage pJD4 with those found in the samples. Blue: *bla*_TEM-1B_; Green: Remaining genes.

We detected Mobile Genetic Elements (MGEs) associated with the *tetM* gene within the plasmid pEP5289, and *bla*_TEM-1B_ gene associated with plasmid pJD4. All samples that yielded positive results for *tetM* or *bla*_TEM-1B_ also exhibited MGEs. The MGEs observed in our genome samples aligned with a reference sequence, revealing a high degree of similarity (Figure 2).

## DISCUSSION

The present study observed a clear phylogenetic grouping of NG STs associated with invasive disease and identified four NG STs which had not been previously described in Brazil. Interestingly, we identified MGEs from the NG genome—plasmids pEP5289 and pJD4—that were potentially associated with increased IGD risk and that patients using immunosuppressive medications might also be at higher risk of IGD. Particularly, we identified potential sub-optimal care for these patients, given the inappropriate or absent antibiotic treatment in a minority of cases and the low frequency of STIs testing.

Said association between the presence of pJD4 and pEP5289 plasmids and IGD occurrence has not been shown previously. Plasmid pJD4 has previously been associated with resistance transfer from beta-lactams to other enterobacteria and *Haemophilus influenzae* ^28,29^. Plasmid pEP5289 has also been associated with the carriage of tetracycline resistance genes and has been found in commensal species of the *Neisseria* genus and other bacteria such as *Kingella denitrifans* and *Eikenella corrodens*, suggesting it functions as a resistance reservoir^30,31^. This possible association between these plasmids and invasiveness may be related to the presence of virulence genes inserted in these MGEs. For example, the *vapD* and *zeta1/2* genes found in plasmid pEP5289 encode two toxins previously described as possible virulence factors in *in vitro* studies^32,33^.

The clustering of invasive samples could indicate that phylogenetic factors may also influence NG pathogenicity. The most prevalent STs observed, 1588 and 1901, were also the most frequently identified in previous Brazilian studies^19^. However, the invasive strains of a previous study conducted by Guglielmino *et al*.^14^ showed no phylogenetic grouping of invasive strains between the two populations analyzed. Moreover, the authors observed a much lower frequency of pJD4 and pEP5289 plasmids among invasive strains compared with our findings.

These findings suggest the existence of other factors related to the pathogen or host that may explain IGD occurrence in that study. The association found between immunosuppression and susceptibility and IGD occurrence had previously been suggested in different immunosuppression contexts. SLE patients using other immunosuppressants and patients with deficiencies in the complement system have already been associated in case series with greater susceptibility to IGD^12,34,35^. These findings highlight the likely influence of host-associated factors on IGD risk, and not NG virulence itself.

The frequency of NG strains in Brazil has been previously described^16,18,19^ using WGS and MLST. These analyses enable evaluating the circulation of genotypic resistance markers and predominant STs, and predict the introduction of international strains with reduced susceptibility to antimicrobials. In the most recent analysis prior to our study, 1901 and 1588 were the predominant STs in Brazil, corresponding to over 30% of strains analyzed^19^. In our study, 48% of specimens belonged to these STs.

A concerning fact is the significant percentage of IGD patients who were not tested for other STIs such as HIV, hepatitis and syphilis. This finding highlights missed opportunities for improving patient care in our institution. Testing for other STIs at the time of IGD diagnosis is a recommended procedure in the STIs management protocol issues by the Brazilian Ministry of Health^36^. STI testing is important for a two-fold reason: first, gonorrhea may increase the risk of acquiring other STIs; second, STI co-infections and associated morbidities are common^37,38^.

Another concerning finding regarding patient care was evidence of sub-optimal or lack of treatment. Three patients did not receive ceftriaxone, two of whom were administered ciprofloxacin monotherapy. Ceftriaxone is recommended by the Ministry of Health^36^ for treating NG in Brazil for all clinical presentations given high resistance rates to fluoroquinolones and macrolides^18^. Moreover, four patients were discharged without any antibiotic prescription, most likely due to prolonged waiting times in urgent care. Improving treatment management of NG patients in this setting is a priority.

Our findings emphasize the need for genotypic surveillance studies of NG isolates beyond antimicrobial resistance profiles, and further studies with a larger and more diverse sample size to confirm such findings. The causality between the presence of pathogen genetic factors and virulence could also be better assessed by *in vitro* studies or animal models, both lacking in this context.

Regarding study limitations, the small sample size and evaluation of patients from a single center may compromise the internal and external validity of our findings, respectively. Evaluation of noninvasive gonococcal infection cases using only patients with positive culture may represent a selection bias, since this subgroup may not be representative of all non-IGD cases. However, to our knowledge this is the first Brazilian study to evaluate genotypic characteristics of NG isolates associated with IGD.

## CONCLUSION

Our results revealed that invasive strains of NG in our sample were genotypically clustered and harbored two plasmids which could be associated with invasive disease, evincing the need for more NG surveillance projects in Brazil to better ascertain these findings.

## Data Availability

The anonymized dataset generated during this study is available from the corresponding author upon reasonable request.
